# Biomarkers in Infectious Diseases

**DOI:** 10.1155/2018/8509127

**Published:** 2018-06-20

**Authors:** Hyundoo Hwang, Boo-Young Hwang, Juan Bueno

**Affiliations:** ^1^BBB Inc., Seoul, Republic of Korea; ^2^Pusan National University Hospital, Busan, Republic of Korea; ^3^Fundación Centro de Investigación y Biotecnología de la Biodiversidad (BIOLABB), Armenia, Colombia

Infectious diseases are categorized as illnesses caused by pathogenic microorganisms such as viruses, bacteria, parasites, or fungi. Such diseases have been major threat worldwide and have a great impact on public health and the world economy. Among different types of infectious diseases, HIV, tuberculosis, and malaria are known as the leading causes of deaths globally. Furthermore, varied types of neglected tropical diseases, such as Chagas disease, dengue, yellow fever, West Nile, Japanese encephalitis, and chikungunya, are also considered to be major global threats. Although such diseases emerge in tropical and subtropical regions, the risk of these infectious illnesses can be worldwide because of global economy and migration. With more than half of the world population at risk of such fatal illnesses, infectious diseases are classified among the most dangerous threats to the society. Fortunately, detection of such infections in the early stages is estimated to significantly reduce the mortality rate. During the past decades, the development of universal and reliable methods to detect biomarkers for diagnostics and prognostics of the infectious diseases and the search for highly specific and sensitive biomarkers have been the most important challenges. The search and discovery of new biomarkers become necessary in infectious diseases in order to determine endpoints, predict the clinical outcome to therapy, and allow the development of new drugs [1].

In this horizon, the search for the ideal biomarkers in infectious diseases (with high sensitivity, specificity, and predictive capacity) must be focused towards detection and identification of the infectious agent, monitoring of the clinical response, and orienting the duration of the treatment, such as the case of procalcitonin (PCT) assay, that can discriminate between a viral and a bacterial infection and has been approved by Food and Drug Administration [2]. Also, the description of new biomarkers requires the development of reproducible diagnostic methods that have accuracy in samples such as blood, sputum, urine, and cerebrospinal fluid [3]. Finally, by virtue of the above, the physician requires robust, reproducible, and automated methods capable of being used within the clinical consultation, in order to give an adequate prescription and an optimized use of the medicines.

In this special issue, we have assembled eight manuscripts identifying biomarkers for infectious diseases including infectious encephalopathy, dengue fever, Kawasaki disease, nephropathia epidemica, and tuberculosis. In the following pages, four research articles identifying serum protein markers for infectious diseases are included. An infectious disease marker that can be detected by a noninvasive urine test is suggested. Also, this special issue includes one and two research articles suggesting metabolic and genetic biomarkers for diagnosis of sepsis, respectively.

Y. Fujii et al. suggest PCT and the ratio of PCT and C-reactive protein (CRP) in serum as biomarkers for the auxiliary diagnosis of acute encephalopathy with biphasic seizures and late reduced diffusion (AESD). AESD is an epilepsy syndrome, which has been known to be associated with infection, most prevalent in East Asia. AESD is characterized by a febrile seizure followed by a cluster of complex partial seizures for several days and reduced diffusion in the frontal and frontoparietal subcortical white matter in magnetic resonance imaging.

M. Vucur et al. demonstrate that serum levels of mixed lineage kinase domain-like protein (MLKL) after three days of intensive care unit (ICU) treatment can be used as a biomarker for prognosis of critically ill and septic patients. MLKL has been known as the key driver of necroptotic cell death. The researchers found that patients with high serum MLKL levels on day three had a significantly impaired survival at the ICU or overall as compared to those with low MLKL. They also found that serum MLKL concentrations correlate with organ failure markers in critically ill and septic patients.

J. Huang et al. investigated cytokine profiles in serum of patients infected with dengue viruses during the Guangdong outbreak in 2014, in which more than 50000 of dengue fever cases were reported and 6 patients died. They found that the levels of CCL17 and CXCL5 were significantly lower than the controls, while several proinflammatory cytokines such as CXCL9, IP-10, CXCL11, IL-8, and IL-10 were highly upregulated in the patients after dengue infection. These results determine the association of clinical routine indexes and the inflammatory cytokines and would be useful to understand the interplay between the virus and the host responses during the acute stage of dengue infection.

S. H. Lee et al. investigated the age-stratified cutoff values of serum N-terminal prohormone of brain natriuretic peptide (NT-proBNP) for the Kawasaki disease patients classified into four subgroups by age (<6 months, 6–12 months, 12–24 months, and >24 months). NT-proBNP has been known as a biomarker for diagnosing Kawasaki disease. However, as the normal range of NT-proBNP widely varies with age, applying the same cutoff value of NT-proBNP to each patient regardless of age would be unreasonable. Therefore, the study by S.H. Lee et al. would provide useful information to apply the serum NT-proBNP level to diagnose Kawasaki disease in patients with different ages to distinguish those conditions from simple febrile illness.

Identifying urinary biomarkers is also very important because they offer more specificity for events in the kidney. E. V. Martynova et al. offer urinary clusterin as a biomarker of early- and late-stage nephropathia epidemica, which is a type of haemorrhagic fever with renal syndrome caused by Puumala virus infection. Nephropathia epidemica is characterized by renal dysfunction which progresses through several stages. The disease progression has been monitored by measuring the levels of blood urea nitrogen (BUN) and creatinine in serum, which reflect renal performance. According to E. V. Martynova et al., however, changes in serum creatinine and BUN concentrations primarily indicate the presence of changes in filtration capacity and, thus, are not always reflective of tissue injury. Therefore, there is a need for an alternative noninvasive method to assess kidney performance in nephropathia epidemica cases. In this study, they found that clusterin is upregulated in urine at the early and late phases of nephropathia epidemica.

M. Lappalainen et al. performed a nontargeted metabolomic profiling to find early diagnostic markers in febrile neutropenia. They found that androsterone/5*α*-dihydrotestosterone sulfate (ADTS/DHTS), citruline, and a fragment of phosphatidylethanolamines can be applied to discriminate patients of febrile neutropenia with complicated and noncomplicated course, based on the significant metabolic features. In particular, ADTS/DHTS shows a strong correlation with plasma CRP and PCT, which are widely used biomarkers in febrile neutropenia.

Genetic markers are also very useful for diagnosing infectious diseases. S. Huang et al. examined expression of long noncoding RNA nuclear-enriched abundant transcript 1 *(NEAT1)* in peripheral blood mononuclear cells in patients with sepsis to explore its diagnostic value and clinical significance in sepsis. *NEAT1* has been known as an important regulator in cancers, as well as in infectious diseases, such as HIV, hantavirus, and Zika virus. In this study, S. Huang et al. first discovered upregulated *NEAT1* expression in patients with sepsis, indicating an association between *NEAT1* and immune dysfunction in sepsis. They suggest *NEAT1* as a potential molecular marker for early diagnosis of sepsis.

Tuberculosis, caused by *Mycobacterium tuberculosis* infection, is also one of the leading causes of death in the world. There were around 1.7 million deaths related to tuberculosis in 2016. Human *speckled 110 (SP110)* gene, which is the closest homology to the mouse intracellular pathogen resistance 1 *(Ipr1)* gene in mouse, mediating innate immunity in mouse TB models, is thought to be associated with tuberculosis susceptibility. Tumor necrosis factor-*α* (TNF-*α*) has also been known as a key player in host resistance to tuberculosis infection. Y. Zhou et al. investigated the influence and difference of single nucleotide polymorphisms in *SP110* and *TNF-α* genes in pulmonary and spinal tuberculosis patients in southern China.

Overall, the research articles in this special issue provide various perspectives on the research in biomarkers of infectious diseases. This special issue is aimed at promoting communication among researchers and broadening our knowledge on biomarkers of infectious diseases. We would like to thank all the authors as well as the reviewers who participated in the elaboration of this special issue.

*Hyundoo Hwang* *Boo-Young Hwang* *Juan Bueno*
